# Review of Lung Particle Overload, Rat Lung Cancer, and the Conclusions of the Edinburgh Expert Panel—It's Time to Revisit Cancer Hazard Classifications for Titanium Dioxide and Carbon Black

**DOI:** 10.3389/fpubh.2022.907318

**Published:** 2022-07-28

**Authors:** Kevin E. Driscoll

**Affiliations:** Ernest Mario School of Pharmacy, Rutgers University, Piscataway, NJ, United States

**Keywords:** lung overload, titanium dioxide, carbon black, rat lung cancer, hazard classification

## Abstract

Chronic inhalation of titanium dioxide or carbon black by rats at concentrations which overload lung particle clearance can result in lung cancer. Based on this rat lung response, IARC, NIOSH, and ECHA classified titanium dioxide, and IARC classified carbon black, as potential human carcinogens. These classifications have been questioned based on an extensive data base demonstrating: the rat lung cancer occurred only under conditions of extreme lung particle overload; the lung cancer response in rats has not been seen in other animal species; and studies in titanium dioxide and carbon black exposed human populations have not shown an increased incidence of cancer. In 2019 an international panel of science and regulatory experts was convened to document the state of the science on lung particle overload and rat lung cancer after exposure to poorly soluble low toxicity particles. Regarding hazard identification, the expert panel concluded, in the absence of supporting data from other species, lung particle overload-associated rat lung cancer does not imply a cancer hazard for humans. Regarding high to low dose extrapolation, the expert panel concluded rat lung tumors occurring only under conditions of lung particle overload are not relevant to humans exposed under non-overloading conditions. The conclusions of the Edinburgh Expert Panel directly conflict with IARC, ECHA and NIOSH's extrapolation of lung particle overload associated rat lung cancer to hazard for humans. The hazard classifications for titanium dioxide and carbon black inhalation should be assessed considering the state-of-the-science on lung particle overload and rat lung cancer.

## Introduction

### Lung Particle Overload

The term “lung particle overload” refers to impairment of particle clearance from the deep lung after inhalation of high concentrations of poorly solubility, low toxicity materials exemplified by titanium dioxide and carbon black. The impairment of particle clearance under these circumstances was proposed by Morrow (1) to be due to the physical loading of macrophages with the consequent loss of cell mobility. Subsequent research has supported impaired macrophage mobility as a contributing factor to lung particle overload and implicated other mechanisms including translocation of particles to the interstitium and lung lymphatics (2, 3). Critical to the definition of “lung particle overload” is it applicability only to materials of low inherent toxicity which differentiates lung particle overload from particle clearance impairment caused by inherently toxic materials which can directly damage macrophages or other lung cells, and in this way reduce particle clearance. Impairment of clearance by inherently toxic materials reflects a different adverse outcome pathway (AOP) from that described by the term “lung particle overload.”

### Rat Lung Cancer and Lung Particle Overload

Over three decades ago, Lee et al. (4) reported chronic exposure of rats to high concentrations of titanium dioxide resulted in lung cancer. This study involved exposure to 10, 50 and 250 mg/m^3^ of respirable particles with lung cancer observed only for the 250 mg/m^3^ exposed rats. Following the Lee et al. (4) publication, additional chronic inhalation studies have reported lung cancer in rats exposed to titanium dioxide and other poorly soluble materials considered to be of low inherent toxicity (summarized in Table 1).

**Table 1:** Chronic inhalation studies in rats producing lung cancer.

**Material**	**Concentration**	**Lung**	**Lung**	**References**
	**(mg/m^**3**^)**	**Burden (mg)**	**Cancer**	
Carbon Black	11.6	43.8	Yes	(5)
Carbon Black	2.5	21.0	Yes	(6)
	6.5	38.5	Yes	
Talc	6	9.7^*^	No	(7)
	18	26.7^*^	Yes	
Titanium Dioxide	10	39.2	Yes	(5)
Titanium Dioxide	10	25.5	No	(4)
	50	124.0	No	
	250	665	Yes	

* *Lung burden normalized to air control lung weight (g)*.

There now exists an extensive toxicology data base in rats and other species on the lung response to poorly soluble, low toxicity particles which has implications for the human relevance of the rat lung cancer response. The following are key findings:

- Rat lung cancer after inhalation of poorly soluble low toxicity particles occurs only under exposure conditions which overload macrophage-mediated particle clearance i.e., cause lung particle overload (3, 8–10).
- A consequence of overloading clearance is a build-up of particulate material in the lung disproportionate to exposures which do not overload lung particle clearance (1, 11).
- In addition to lung cancer, lung particle overload in rats is associated with pulmonary inflammation; lung epithelial cell hyperplasia and metaplasia; and pulmonary fibrosis. These non-neoplastic responses precede development of lung cancer and occur at exposure levels not causing cancer (4–7, 12).
- Lung cancer has not been observed in other animal species (i.e., mice and hamsters) after chronic inhalation exposure to the materials in Table 1 under conditions of lung particle overload (5–7, 13, 14).
- Epidemiology studies have not demonstrated a significant increase in lung cancer after exposure to the materials in Table 1 (15–18).

### Hazard Classification of Titanium Dioxide and Carbon Black

Several organizations have characterized the health hazards associated with titanium dioxide and carbon black inhalation, including IARC, ECHA, and NIOSH. The outcomes of these evaluations are summarized below.

#### IARC

IARC classified titanium dioxide and carbon black as “possibly carcinogenic to humans” based on lung cancer occurring in rats (16). The IARC review reported there was no convincing evidence of cancer in humans exposed to these materials. In the same IARC monograph, talc was determined to be not classifiable as to its carcinogenicity based on limited data in animals (e.g., a single chronic inhalation study in rats which showed lung cancer under lung clearance overload and a chronic inhalation study in mice which was determined to be negative for cancer) and, inadequate evidence in humans for talc not containing asbestos or asbestiform fibers.

#### ECHA

ECHA's Committee for Risk Assessment (RAC) recommended titanium dioxide be classified as suspected of causing lung cancer through the inhalation route (19). This assessment was based on a chronic titanium dioxide inhalation study in rats which, in the words of the study investigators (5): “*induced lung tumours in rats under conditions of marked particle loading in the lung*.” RAC concluded human data do not support an association between occupational exposure to titanium dioxide and risk of lung cancer. ECHA subsequently adopted the RAC recommendation on titanium dioxide hazard. Observations from the RAC documentation on titanium dioxide include:

- RAC did not use the Lee et al. (4) study to support its classification, concluding “*these exposure conditions represent excessive exposure which invalidates the results of the Lee et al*. *(**4**)* *study on their own for classification purposes*.” RAC noted such a marked condition of overload should not be a determining factor on classification of titanium dioxide.
- RAC's classification of titanium dioxide relied on “*selected carcinogenicity data for poorly soluble low toxicity particles as supporting evidence*.” RAC did not provide a definition of poorly soluble low toxicity particles, although carbon black was discussed in this context.
- In selecting relevant studies for classification RAC chose not to follow OECD Guidance Document 116 (20) which recommended safety evaluation not be based on experimental exposure levels of particles resulting in an elimination half-time of ~1 year due to lung overload. RAC's rational for not following OECD guidance was OECD did not provide a justification for the 1 year half-time. Of note, the titanium dioxide clearance half-time in the rat study RAC used for classification was reported to be 500 days (2).
- RAC discussed coal dust as an example of human exposure to a poorly soluble low toxicity material which supports the potential human relevance of rat lung overload associated cancer (19).

#### NIOSH

NIOSH differentiated their cancer hazard classification based on the particle size of titanium dioxide (21). Ultrafine titanium dioxide (<100 nm diameter) was classified as a potential occupational carcinogen based on the Heinrich et al. (5) inhalation study in rats. In contrast, NIOSH concluded for fine size titanium dioxide (>100 nm diameter) there were insufficient data to classify as to carcinogenicity. NIOSH recommended separate exposure limits (RELS) for ultrafine (0.3 mg/m^3^) and fine size titanium dioxide (2.4 mg/m^3^). The RELS were based on an extrapolation of the rat inhalation data to humans using particle surface area as the dose metric which several studies have suggested is a more relevant dose metric for the cancer response in the rat studies (22, 23). Other key observations from the NIOSH discussion of the titanium dioxide inhalation hazard include:

- NIOSH disregarded the Lee et al. (4) study, stating: “*because this dose is considered to be significantly higher than currently accepted inhalation toxicology practice* *(**24**), NIOSH concluded that the response at such a high dose should not be used in making its hazard identification*”.
- NIOSH analyzed the rat lung cancer and exposure dose relationships for poorly soluble low toxicity materials and concluded the cancer risk of titanium dioxide inhalation is most closely related to the surface area dose of the particulate.
- NIOSH concluded the adverse effects of inhaling titanium dioxide may not be material-specific but due to a generic effect of poorly soluble low toxicity materials. While not providing a definition of poorly soluble low toxicity, NIOSH listed materials in this group as including titanium dioxide, BaSO_4_, carbon black, toner, and coal dust.
- NIOSH discussed coal dust as an example of poorly soluble low toxicity particulate exposure in humans. NIOSH cited data on lung burden in coal miners as supporting the human relevance of the titanium dioxide lung burdens and lung cancer findings occurring in rats under lung clearance overload.

### Edinburgh Expert Workshop on the Hazards and Risks of Poorly Soluble Low Toxicity Particles

In, a panel of scientists and regulators with extensive expertise on particle inhalation toxicology and risk assessment was convened to document the state-of-the science on the hazards and risks of inhaled of poorly soluble, low toxicity materials. This workshop also included observers from government and industry representing important stakeholders on the topics being considered. Details on the experts, the observers, the charges to the panel and the outcomes can be found in Driscoll and Borm (25). For convenience, the expert panel members and observers are summarized in Tables 2A,B.

**Table 2A:** Edinburg Workshop Expert Panelists.

Armelle Baeza-Squiban, Ph.D.	Professor, Functional and Adaptive Biology, Paris Diderot, University
Flemming Cassee, Ph.D.	National Institute for Public Health and the Environment, Netherlands
Rodger Duffin, Ph.D., MRC Path, FRSB	Reader in Respiratory Medicine, University of Edinburgh
Tom Gebel, Prof. Dr.	German Federal Institute for Occupational Safety and Health
Helmut Greim, M.D.	Technical University Munich
Uwe Heinrich, Dr. rer. nat.	Toxicology and Aerosol Research, Medizinische Hochschule, Hannover
Wolfgang G. Kreyling, Dr. rer. Nat.	German Research Center for Environmental Health
Robert Landsiedel, Dr. rer. nat. habil.	BASF SE. Experimental Toxicology and Ecology
Len Levy, Ph.D.	Cranfield University
Dominique Lison, M.D. Ph.D.	Louvain Centre for Toxicology and Applied Pharmacology (LTAP)
Fred J. Miller., Ph.D.	Inhalation Toxicology Division, US EPA; Fred Miller and Associates
Günter Oberdörster, Prof	Dept of Environ. Medicine, University of Rochester School of Medicine and Dentistry
Lang Tran, Ph.D.	Institute of Occupational Medicine, Edinburgh, UK
David B Warheit, Ph.D.	Warheit Scientific LLC
Mei Yong, Dr. rer. Medic.	Inst. for Occup. Epidemiology and Risk Assessment, Evonik Technology and Infrastructure

**Table 2B:** Edinburgh Workshop Observers.

Damjana Drobne	University of Ljubljana, Biotechnical Faculty (SL) *Ad hoc* CARACAL sub-group on ATPs to CLP classification of TiO_2_ and mixtures.
Craig Boreiko	Consultant to Antimony Association
Fiona Murphy	Herriot Watt University- Edinburgh; Member of the EU GRACIOUS Consortium
Annie Jarabek	U.S. EPA, National Center for Environmental Assessment (NCEA)
Terry Gordon	New York University School of Medicine, ACGIH TLV Committee
Klaus Kamps	Unifrax; Chair of Eurometaux REACH working group
Roger Battersby	EBRC Consulting
Frank Luetzenkirchen	Quarzwerke GmbH, Frechen, Germany (DE); IMA-Europe: Chairman IMA Technical Board
Robert McCunney	Harvard Medical School; Consultant to International Carbon Black Association
David Lockley	Product Defense and Toxicology Manager, Venator Corp; Chair of Scientific Committee and CLH TF, TDMA
Sue Hubbard	Consultant Regulatory Toxicologist Sah Co., Ltd. (UK); Member of Iron Platform
Andrew Smith	Health and Safety Executive (UK); Chemicals Regulation Division, Team leader: REACH-CLP-PIC; Member of ECHA's Risk Assessment Committee
Tim Bowmer	European Chemicals Agency (ECHA); Chairman of the Committee for Risk Assessment
Ari Karjalainen	European Chemicals Agency (ECHA), Unit C1—Hazard I
Yufanyi Ngiewih	Orion Engineered Carbons GmbH; ICBA Scientific Advisory Board

The Edinburgh Expert Panel reached agreement on the state-of-the science for several topics relevant to the application of inhalation toxicology data for hazard identification and risk assessment of titanium dioxide, carbon black and other materials characterized as poorly soluble and low toxicity. Key areas of consensus included:

- In the absence of supporting data from other species, particle overload-associated lung cancer in rats should not be extrapolated to human lung cancer hazard.
- Lung cancer in rats occurring only under conditions of lung particle overload does not imply a cancer hazard for humans under non-overloading exposures.
- Materials with unknown toxicological profiles should NOT be grouped with poorly soluble low toxicity materials without data demonstrating comparable low solubility and low toxicity.
- Increased particle retention resulting from large lung burdens of low toxicity materials is distinct from increased particle retention due to the inherent cytotoxicity of particles (e.g., quartz).

## Discussion

The Edinburgh Expert Panel's conclusions on the state of the science regarding extrapolating hazards of poorly soluble low toxicity materials have implications for the cancer hazard classifications developed previously for titanium dioxide (by IARC, ECHA, NIOSH) and for carbon black (by IARC).

### Cancer Hazard Classification Based on Inhalation Data From Rats

In their reviews of the titanium dioxide data base IARC, ECHA and NIOSH made several general observations which can be summarized as follows: lung cancer in rats was associated with the overload of lung particle clearance; lung cancer was not observed in other animal species exposed chronically by inhalation; and there was no convincing evidence of lung cancer in humans. In the end, IARC, ECHA, and NIOSH rendered classifications of titanium dioxide based solely on studies in rats in which lung cancer occurred under conditions of lung particle overload. IARC based its concern classification of titanium dioxide solely on studies in rats in which cancer only occurred under lung particle overload (4, 5). Similarly, in its evaluation of carbon black, IARC based the classification solely on studies demonstrating rat lung cancer under conditions of lung particle overload. NIOSH and ECHA disregarded the Lee et al. (4) study assessing the exposures as to excessive and based their cancer classification on a single study (5). Considering NIOSH and ECHA's basis for rejection of the Lee et al. study (4), questions can be raised as to why these groups accepted of the Heinrich et al. (5) given the rat lung cancer occurred under conditions described by the study investigators as “severe dust overloading,” with a titanium dioxide lung clearance halftime of 500 days, and no reversibility of lung clearance (2, 5). The basis of the IARC, ECHA and NIOSH classification conflict with the more recent assessment of the state-of-the science by the Edinburgh Expert Panel regarding the extrapolation of rat lung cancer outcomes, observed on under lung particle overload and with no supporting data from other species.

### Coal Dust Is Not a Suitable Reference for Titanium Dioxide

In their classification of titanium dioxide, both ECHA and NIOSH reference coal-dust exposed workers to support the human relevance of the lung burdens in rats causing lung cancer. First, it should be noted that a preponderance of the epidemiology data does not support an association between coal dust exposure and lung cancer or lung clearance impairment (26, 27). Regarding poorly soluble low toxicity dusts, neither NIOSH nor RAC provide a definition, however, NIOSH lists coal along with titanium dioxide, BaSO_4_, carbon black, toner as a group they consider to be poorly soluble and low toxicity. A scientific issue regarding use of coal dust lung burden data to support the human relevance of the rat cancer after titanium dioxide, is coal is quite different toxicologically. Briefly, coal dust can contain significant amounts of quartz; trace metals such as boron, cadmium, copper, nickel, iron, and zinc; as well as in organic minerals (27). Quartz is a well-established lung toxin, directly toxic to macrophages and other lung cells (27). Regarding trace metals, studies on coal have demonstrated the iron present generates reactive oxygen species which contributes to coal dust toxicity to lung macrophages and epithelial cells (28–30). Moreover, in studies directly comparing the effects of coal dust and titanium dioxide on human macrophages, coal dust but not titanium dioxide, was shown to activate macrophage release of the potent proinflammatory cytokines tumor necrosis factor α and interleukin 6 which can contribute to lung disease (31). As concluded by the Edinburgh Expert Panel, before grouping materials for safety considerations, there needs to be data demonstrating similarity in solubility and toxicity profiles. Existing data on coal dust demonstrates it is clearly different from titanium dioxide in its inherent toxicity. On a scientific basis such differences arguably preclude the use of coal dust exposure and lung burdens as a surrogate for titanium dioxide, carbon black and other comparable poorly soluble low toxicity dusts.

### Does Lung Particle Overload Occur in Humans?

The expert panel agreed that lung particle overload has been demonstrated in all laboratory animal species evaluated. As such, there was agreement lung particle overload could occur in humans, however, there was not agreement on whether this has been proven (25). In this respect, it is noteworthy that even in coal miners with extremely high lung burdens of coal dust, which is inherently more toxic than titanium dioxide or carbon black, significant prolongation of lung particle clearance has not been demonstrated (26, 32, 33). This raises the question: if lung particle overload with its various sequelae can occur in humans, what magnitude of lung exposure to truly low solubility, low toxicity materials (i.e., titanium dioxide and carbon black) would be required? It can be anticipated that the magnitude and duration of such hypothetical exposures would have no relevance to occupational exposures reported for titanium dioxide and carbon black (26, 33).

### Summary and Recommendations

The finding that chronic inhalation of titanium dioxide or carbon black results in lung cancer in rats but not in other species and that the rat lung cancer occurs only under conditions of extreme lung particle overload has raised questions on the relevance of overload-associated rat lung cancer to human hazard (8, 11, 13, 34, 35). Despite significant questions on the predictiveness of the rat lung cancer response, IARC, NIOSH and ECHA identified titanium dioxide as a cancer hazard for humans based solely on rat lung cancer. IARC made a similar classification of carbon black, again based solely on lung cancer in rats occurring under lung particle overload.

In 2019, a panel of scientists and regulators expert in inhalation toxicology and risk assessment was convened at the University of Edinburgh to document the state-of-the-science on rat lung cancer and lung particle overload. Regarding hazard identification, the expert panel concluded that in the absence of supporting data from other species, lung particle overload-associated rat lung cancer does not imply a cancer hazard for humans. In the context of high to low dose extrapolation, the expert panel concluded rat lung tumors occurring only under conditions of lung particle overload are not relevant to humans under non-overloading exposures to poorly soluble low toxicity materials. Hazard identification represents an important activity to ensure public health; however, such identification needs to take full account of the state-of-the-science and be updated as scientific understanding advances. In this respect, the conclusions of the Edinburgh expert panel call for a reassessment of the cancer hazard classifications on titanium dioxide and carbon black taking into full account the current scientific understanding of lung particle overload, rat lung cancer and species differences in lung cancer response to poorly soluble, low toxicity materials.

## Author Contributions

The author confirms being the sole contributor of this work and has approved it for publication.

## Conflict of Interest

The author declares that the research was conducted in the absence of any commercial or financial relationships that could be construed as a potential conflict of interest.

## Publisher's Note

All claims expressed in this article are solely those of the authors and do not necessarily represent those of their affiliated organizations, or those of the publisher, the editors and the reviewers. Any product that may be evaluated in this article, or claim that may be made by its manufacturer, is not guaranteed or endorsed by the publisher.
